# Social acceptance of genetic engineering technology

**DOI:** 10.1371/journal.pone.0290070

**Published:** 2023-08-16

**Authors:** Katherine E. Koralesky, Lara V. Sirovica, Jillian Hendricks, Katelyn E. Mills, Marina A. G. von Keyserlingk, Daniel M. Weary

**Affiliations:** Faculty of Land and Food Systems, Animal Welfare Program, The University of British Columbia, Vancouver. British Columbia, Canada; University of Florida, UNITED STATES

## Abstract

Genetic engineering of animals has been proposed to address societal problems, but public acceptance of the use of this technology is unclear. Previous work has shown that the source of information proposing the technology (e.g. companies, universities), the term used to describe the technology (e.g. genome editing, genetic modification), and the genetic engineering application (e.g. different food products) affects technology acceptance. We conducted three mixed-method surveys and used a causal trust-acceptability model to understand social acceptance of genetic engineering (GE) by investigating 1) the source of information proposing the technology, 2) the term used to describe the technology, and 3) the GE application for farm animals proposed. Further, participants expressed their understanding of technology using a range of terms interchangeably, all describing technology used to change an organism’s DNA. We used structural equation modelling and confirmed model fit for each survey. In each survey, perceptions of benefit had the greatest effect on acceptance. Following our hypothesized model, social trust had an indirect influence on acceptance through similar effects of perceived benefit and perceived risk. Additional quantitative analysis showed that the source of information and technology term had little to no effect on acceptance. Applications involving animals were perceived as less beneficial than a plant application, and an application for increased cattle muscle growth was perceived as more risky than a plant application. When assessing the acceptability of applications participants considered impacts on plants, animals, and people, trust in actors and technologies, and weighed benefits and drawbacks of GE. Future work should consider how to best measure acceptability of GE for animals, consider contextual factors and consider the use of inductive frameworks.

## Introduction

A range of technologies can be used to change the genetics of an organism (referred to as gene editing, genetic modification, etc.; collectively referred to here as genetic engineering or GE). Proponents of these technologies have suggested that these can be used to address a variety of societal and environmental challenges, including human health and environmental concerns [1,2]. In farming, applications have been proposed to improve disease resistance in animals, reduce agricultural pollution and improve animal welfare [3,4].

Some research has focused on what GE means for the animals involved and what people consider to be acceptable for animals. Early research focused on the negative effects on animals such as genetic disorders and development abnormalities [e.g. 5] and the role humans ought to play in modifying animal genomes [i.e. “playing God”; 6]. However, much of this scholarship took place before the development of technologies that can target specific genes [e.g. CRISPR/Cas9; 7]; such technologies are considered more precise, changing some ethical considerations [8]. In response to the growing discussion regarding ethical considerations about this technology, [9] conducted a systematic review of reasons given for and against gene editing in non-human animals. Their findings indicate that the reasons against gene editing included concerns about environmental safety, animal health and public acceptability, while the most cited reason in favor of this approach was the potential to improve human health (although this improvement focused on the application of gene drives to control vector-borne diseases).

A large body of research now exists on public attitudes specifically towards foods resulting from GE. Researchers have reviewed behavioral processes that can lead to acceptance, including perceptions of risk and benefit, trust, knowledge, valuation and purchasing decisions [10,11]. For example, one review reported that genetically modified (GM) meat was perceived as less acceptable than GM oil [11]. Others have evaluated consumer and societal attitudes toward genetically engineered food [12] and, like [10], identified that risk and benefit perceptions and intention to purchase as well as awareness, beliefs, concerns and emotions play a role in determining attitudes. In another instance, [13] also reviewed risk, benefit and trust perceptions, but also considered perceptions of “naturalness” and technology use in food. Finally, [14] compared a range of novel food technologies (including GE, food irradiation, nanotechnology) and reported that use of the term “bioactive” to describe technologies led to “uncomfortable” perceptions of the technology. Individual differences, such as levels of food neophobia, disgust and cultural values have also been reported to account for differing perceptions of novel food technologies [15].

Understanding ways that people understand technologies has also been explored, including the role of the source of information proposing the technology. The role of trust in institutions that are developing or using technologies has also been explored, including scientists and researchers at universities, pharmaceutical companies, agricultural companies and food companies [16,17]. For example, [18] reported that there was less trust in information on GE foods from private industry, environmental or consumer groups compared to independent third-party sources. In a survey measuring the level of support for GE products created by 14 different organizations, [19] reported least support for a “chemical company” and most support for a “university.” Thus, the source conveying the information about the technology, developing the technology, or using the technology appears to be important in people’s assessment of different technologies.

Some work has examined how people perceive the terms used to describe GE technologies. Commonly used terms include “genetic modification”, “gene editing”, “genetic manipulation” and “genetic engineering” [20–22]. Findings vary, but gene editing is generally reported to be perceived more positively than genetic modification [22,23].

People also appear to vary in their support for GE based on the perceived benefits of specific applications. Some applications directly benefit human welfare (e.g., vaccine development [24]), and applications that increase human disease resistance [25] or improve human health and medicine [26] have been judged as more morally right or acceptable compared to plant and animal applications. Genetically modified crops, for example herbicide-tolerant and insect-resistant cotton and corn increased profits, yields and reduced chemical pesticide use [27]. While some suggest that such benefits may improve public trust in the technology [27], others consider that initial promises of the technology (increased global food security, sustainability) did not come to fruition and rather further engrained monocultures and enabled large farms to reduce labor costs [28].

Regarding farm animals, in three studies about genetically engineered hornless cattle [29–31], at least some participants were more willing to accept the application if they felt that it benefited the animal (in this case avoiding the need for painful surgery to remove horn buds in calves). Others have examined differences between plant and animal applications. For example, [32] reported that survey participants considered gene editing of plants to be less concerning than gene editing of livestock. Among other measures, [25] found that participants were more supportive of GE applications targeting disease resistance in plants than in animals, and both applications were more supported than those intended to improve quality or quantity of products. Thus, GE applications seem to be more acceptable when they are used to address issues of public good and less acceptable when they are perceived to primarily benefit private actors, for example improving farm profit by increased production [33].

People may lack adequate knowledge about GE to feel comfortable evaluating whether they find it acceptable or not, and thus rely on social trust (i.e., trust in regulatory and other institutions) when judging risks and benefits [15,16]. Given that we were specifically interested in the social acceptance of GE of animals, we applied a causal trust-acceptability model [following 34]. Specifically, the causal trust-acceptability model seeks to understand the influence of social institutions on acceptance of technology [16,35]. Other technologies have been examined using this well-established model including sources of electrical generation [34], gene recombination technology [36] and genetically modified food [37], but little research has examined animals specifically. Thus, our objective was to understand social acceptance of GE technologies in agricultural contexts with farm animals. We conducted three separate surveys and used structural equation modeling (SEM) to assess model fit. We then investigated the effects of additional contextual factors, primarily the source of information proposing the technology (Survey 1), the term used to describe the technology (Survey 2); and the specific application proposed (Survey 3).

## Materials and methods

### Ethics

The University of British Columbia Behavioral Ethics Board (#H21-00047) approved this study.

### Causal model and survey design

The causal trust-acceptability model includes the latent variables of acceptance, perceived benefit, perceived risk, and social trust. Latent variables are considered to be unobservable constructs and thus must be measured using a series of observed variables, in this case participant responses to individual survey questions (Table 1).

**Table 1:** Likert scale questions adapted from [34]^1^. Brackets are used to designate where we modified wording in Survey One.

**Perceived benefit**	**(1 = low benefit to 5 = high benefit)**
B1 In general, how beneficial do you consider this technology to be for society as a whole?	Not beneficial at all–Very beneficial
B2 In general, how beneficial do you consider this technology to be for you?	Not beneficial at all–Very beneficial
B3 In general, how beneficial do you consider this technology to be for the environment?	Not beneficial at all–Very beneficial
**Perceived risk**	**(1 = low risk to 5 = high risk)**
R1 In general, how risky do you consider this technology to be for society as a whole?	Not risky at all–Very risky
R2 In general, how risky do you consider this technology to be for you?	Not risky at all–Very risky
R3 In general, how risky do you consider this technology to be for the environment?	Not risky at all–Very risky
**Social trust in institutions (reverse scored)** ^2^	**(1 = low social trust to 5 = high social trust)**
T1 I feel confident that the [source of information] has the competence to make good decisions related to this technology	Totally disagree–Totally agree
T2 I feel confident that the [source of information] has the competence to properly assess risks and benefits related to this technology	Totally disagree–Totally agree
T3 I feel confident that the [source of information] has the competence to solve problems related to this technology	Totally disagree–Totally agree
T4 I feel confident that the [source of information] is concerned about safeguarding the interests of the citizens and the environment when it comes to assessing risks and benefits of this technology	Totally disagree–Totally agree
T5 I feel confident that the [source of information] acts without political or private pressures and obligations when assessing this technology	Totally disagree–Totally agree
**Acceptance**	**(1 = low acceptability to 5 = high acceptability)**
A1 In general, how acceptable are the risks associated with this technology for society as a whole?	Unacceptable–Acceptable
A2 In general, how acceptable are the risks associated with this technology for the environment?	Unacceptable–Acceptable
A3 When it comes to the risk-benefit balance for the society as a whole associated with this technology	Risks greatly outweigh benefits–Benefits greatly outweigh risks
A4 When it comes to the risk- benefit balance for the environment associated with this technology	Risks greatly outweigh benefits–Benefits greatly outweigh risks

^1^ We listed scale endpoints following [34].

^2^ Reverse scored to match scoring of other latent variables (social trust was originally scored as high score = low trust).

**Fig 1:**
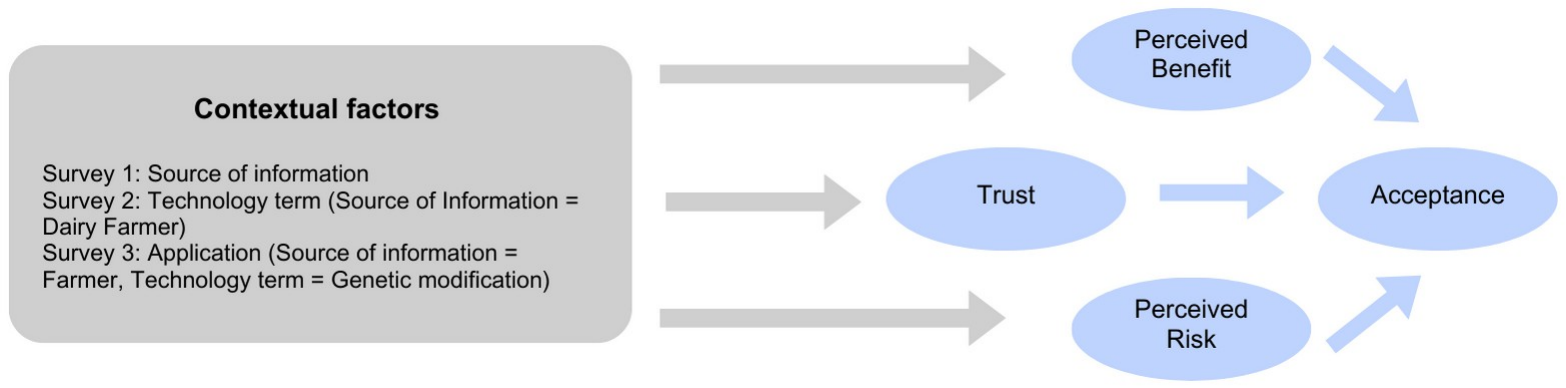
Hypothesized model of social acceptance of GE technology considering contextual factors. Adapted from [16,35,34].

Adapted in part from [34] and [16,35], we created a model (Fig 1) that shows our hypothesized causal trust-acceptability model of the social acceptance of GE technology. Structural equation modeling (also referred to as causal modeling) is useful when investigating relationships between multiple latent variables, as is the case with the social acceptance model used here. We used SEM to assess model fit for both our measurement model (examining how the survey responses relate to the latent variables) and a structural model (examining associations among the latent variables) [38,39]. We tested the effect of treatment and demographic factors, separately for each of the three surveys, on each of the latent variables that our SEM found to be linked to acceptance (i.e., perceived benefit, perceived risk, and social trust). To help better understand participants’ views, we added open-ended questions to each survey [40,41].

### Recruitment

We created each survey in UBC-hosted Qualtrics (Provo, UT, USA) and recruited Canadian and USA participants 18 years of age or older in summer 2021 through Amazon Mechanical Turk crowd sourcing service (MTurk). Each participant provided written consent to take the survey and participant quotas were set based on representative census data from the USA [42] and Canada [43]. We compensated USA participants $1.00 USD. Canadian participants received $1.00 CAD in Survey 1, but the amount was increased to $2.00 CAD in Surveys 2 and 3 to increase recruitment. A copy of each survey and survey responses can be found in the supplementary materials (see Supplemental Material; https://doi.org/10.5683/SP3/NX3LZ9).

### Survey procedures

We randomly assigned participants to treatments. Each treatment presented participants with a short vignette, followed by 5-point Likert scale questions (Table 1) and open-ended questions that varied slightly depending on the contextual factors examined in each survey (i.e. source of information, technology term and application). For example, the vignette in Survey 1 read [adapted from 44]:

Our climate has warmed by 1.7 °C since 1948, leading to an increase in the frequency of very hot days. Cattle are susceptible to heat stress during periods of hot weather.

[Source of information] have proposed gene editing cattle to address this problem; in this way cattle can be made more resistant to heat stress by adding genes from heat-resistant cattle breeds.

Fifteen sources of information (treatments) were assessed: government scientists, dairy industry scientists, university scientists, animal scientists, food scientists, environmental scientists, private industry scientists, dairy processors, dairy farmers, dairy veterinarians, grocery stores, dairy producers, animal advocates, animal humane organizations, and environmental advocates. After participants responded to the Likert-scale questions, they answered two open-ended questions: “How do you understand the term [source of information]?” and “How do you understand the term gene-editing?”

Through preliminary qualitative data analysis, we determined that the term “dairy farmer” was understood well (i.e. participants’ definitions of “dairy farmer” included elements of a dairy farmer’s actual role in society) and neutrally (i.e. participants did not express overly positive or negative attitudes toward dairy farmers), and given our interest in dairy farming, we replaced [source of information] with “dairy farmer” in Survey 2.

For Survey 2, the original vignette was used but the source of information for all treatments was “dairy farmer”, and the technology term varied; specifically, the vignette read: “Dairy farmers have proposed [technology term] cattle to address this problem.*”* Eight technology terms (treatments) were assessed: gene editing, genetic modification, genome editing, genetic engineering, genomic alteration, DNA modification, genetic manipulation, and genetic alteration. After participants responded to the Likert-scale questions, they answered one open-ended question: “How do you understand the term [technology term]?”

Through preliminary qualitative data analysis, we determined that the term “genetic modification” was understood well (i.e. participants’ definitions of “genetic modification” included that it involved changing the genes or DNA of the animal). It was also the most neutral (i.e. participants did not use overly positive or negative words to describe the term genetic modification compared to other terms). We therefore replaced [technology term] with “genetic modification” in Survey 3. For this final survey, we developed six vignettes (treatments), each describing one application of GE technology (Table 2). We used cis-genetic engineering examples following the format of [25] and real-world applications described in [4,45,46]. In addition to responding to the Likert-scale questions, we asked participants one open-ended question: “In a few sentences, please describe how acceptable you think this technology is for the [animals or plants depending on treatment] involved.”

**Table 2:** Survey 3 vignettes describing different genetic engineering applications; each participant was presented with one vignette.

Treatment	Vignette provided to participants
^1^ **Original wording (from Survey 1 and Survey 2): Climate change and cattle heat stress resistance**	Our climate has warmed by 1.7 °C since 1948, leading to an increase in the frequency of very hot days. Cattle are susceptible to heat stress during periods of hot weather. Genetic modification of cattle has been proposed by dairy farmers to address this problem; in this way cattle can be made more resistant to heat stress by adding genes from heat-resistant cattle breeds.
^1^ **New wording: Climate change and cattle heat stress resistance**	Heat stress is an important concern in livestock farming. Cattle are susceptible to heat stress during periods of hot weather. Genetic modification of cattle has been proposed by farmers to address this problem; in this way cattle can be made that are more resistant to heat stress.
**Wheat plant drought resistance**	The risk of disease is an important concern in agriculture. Mildew is an infection that wheat plants can contract. Genetic modification of wheat plants has been proposed by farmers to address this problem; in this way wheat plants can be made that are more resistant to this disease.
**Pig disease resistance**	The risk of disease is an important concern in agriculture. Reproductive and respiratory syndrome is a disease that pigs can contract. Genetic modification of pigs has been proposed by farmers to address this problem; in this way pigs can be made that are more resistant to this disease.
**Allergen-free milk**	Food safety is an important concern in agriculture. Some people have an allergic reaction to cow’s milk. Genetic modification of cattle has been proposed by farmers to address this problem; in this way cattle can be made that produce allergen-free milk.
**Increased muscle growth in cattle**	Productivity is an important concern in agriculture. Slow-growing cattle can be more expensive for farmers to raise. Genetic modification of cattle has been proposed by farmers to address this problem; in this way cattle can be made that are more productive.

^1^These two treatments differing in original and new wording were later combined into one treatment for climate change and cattle heat stress resistance following results of no difference in perceptions between them.

### Data preparation

We prepared data by 1) removing incomplete responses, 2) removing responses in which participants failed the instructed manipulation check [47], and 3) removing responses in which the participants’ qualitative responses were blank or contained non-sensical text [48,49]. In total, 1,522, 1,196 and 1,462 participants completed Surveys 1, 2, and 3, respectively. Participants who failed the attention check (Survey 1 = 78; Survey 2 = 100; Survey 3 = 81) or who wrote nonsensical qualitative answers (Survey 1 = 106; Survey 2 = 16; Survey 3 = 154) were removed. This resulted in a total of 1,338, 1,080 and 1,227 participant responses for analysis in Surveys 1, 2, and 3, respectively.

We assessed the originality of qualitative responses using Turnitin, an online writing assessment tool [50]. Qualitative responses flagged by Turnitin as more than 50% copied from online sources were categorized as copied (i.e. unoriginal). We then read each response to identify copied responses missed by Turnitin. Participants who failed the originality check (Survey 1 = 215; Survey 2 = 177; Survey 3 = 272) were removed, leaving a total of 1,123, 903, and 955 participant responses for qualitative analysis in Surveys 1, 2, and 3, respectively.

### Quantitative data analysis

We used SAS ^®^ OnDemand for Academics (version 9.4, SAS Institute Inc.; SAS models are provided in the Supplemental Materials; https://doi.org/10.5683/SP3/NX3LZ9). Cronbach’s α was used to assess the internal reliability of each series of questions [51]. Cronbach’s α was high for each series of questions in each survey (see Table Cronbach’s α in Supplemental Material; https://doi.org/10.5683/SP3/NX3LZ9). For each survey, we used PROC CALIS in SAS to test model fit using measurement and structural models. We developed models for each survey using path coefficients (factor loadings from the confirmatory factor analysis) for the measurement and structural model and tested overall model fit using the Comparative Fit Index (CFI) and Root Mean Square Error of Approximation (RMSEA). Values of CFI greater than or equal to 0.94 are considered good and values of RMSEA lower than or equal to 0.055 are considered good and between 0.056 and 0.08 are considered adequate [39].

Given the results of our SEM, confirming our predicted model for acceptance for each of the three surveys, we then used general linear models to test the effects of treatment, demographics, and first order interactions between treatment and demographics on each of the latent variables predicting acceptance (i.e. perceived benefit, perceived risk, and social trust). Interaction terms with P > 0.05 in preliminary models were removed; all other variables were retained in the final models.

Demographic variables included: geographic area (rural versus urban), country (Canada versus USA), education (did not graduate high school versus high school graduate versus some college, no degree versus trade degree versus bachelor’s degree versus graduate degree), gender identity (woman versus other), income, and generation (Generation Z (Gen Z) versus Millennial versus Pre-millennial). Gen Z was defined as participants aged ≤ 24 years old, Millennials as participants aged 25 to 40 years old, and Pre-millennials as participants aged ≥ 41 years old as self-reported at the time of taking the survey [52]. The effects of variables with P ≤ 0.05 are discussed for each model below.

Least squares means and standard errors (± SE) from the general linear models are presented for treatments. In Survey 3, a planned comparison between the original wording and the new wording of the climate change and cattle heat stress resistance treatments was run in preliminary models for each latent variable to test whether the difference in wording affected perceptions. We found no effect on acceptance (F_1,1221_ = 0.67; P = 0.41), perceived benefit (F_1,1221_ = 1.27; P = 0.26), perceived risk (F_1,1221_ = 0.82; P = 0.37), or social trust (F_1,1221_ = 0.50; P = 0.48) and so combined responses from the original and new wording treatments. In Survey 3, a planned comparison between treatments involving the plant application and the four treatments involving animal applications was also assessed for the final models on perception of benefit, of risk, and social trust.

### Qualitative data analysis

We used descriptive coding to analyze open-ended responses [53]. This process involves first classifying responses using codes (i.e. words or short phrases that describe the response) and then categorizing codes to develop themes based on the question asked. The third author coded responses in Survey 1 (source of information) and 3 (application). The primary author coded responses in Survey 1 and 2 (technology terms). These authors created codebooks for each dataset and then randomly selected approximately 15% (n = 100) of responses to perform intercoder agreement. After coding independently, they met to compare coding, discuss discrepancies and make clarifications to the codebook before proceeding with analysis. They then performed intercoder reliability twice with each dataset to improve the dependability of qualitative analysis [54]. Participant quotations are identified by country (US, CA), Survey number (1–3) and random participant identification number (e.g. US1_623, CA2_40) (see Supplemental Material; https://doi.org/10.5683/SP3/NX3LZ9).

## Results

Participant demographic information is included in Table 3. Survey completion time (means ± SE) was similar across surveys: Survey 1 (5 min 23 sec ± 10 sec), Survey 2 (4 min 36 sec ± 8 sec), Survey 3 (5 min 33 sec ± 9 sec).

**Table 3:** Descriptive statistics about survey participants including geographic area, country, education, gender identity and generation. The number of participants per treatment for Surveys 1, 2 and 3 are specified in the supplementary materials (see Supplemental Material; https://doi.org/10.5683/SP3/NX3LZ9).

		Survey 1 (n = 1338)		Survey 2 (n = 1080)		Survey 3 (n = 1227)	
**Demographic**	Level	n	%	n	%	n	%
**Geographic area**	Rural	289	21.6	278	25.7	268	21.8
	Urban	1049	78.4	802	74.3	959	78.2
**Country**	Canada	143	10.7	143	13.2	99	8.1
	USA	1195	89.3	937	86.8	1128	91.9
**Education**	Did not graduate high school	12	0.9	5	0.5	2	0.2
	High school graduate	95	7.1	85	7.9	87	7.01
	Some college, no degree	244	18.2	175	16.2	159	13.0
	Trade degree	70	5.2	56	5.2	68	5.5
	Bachelor degree	702	52.5	555	51.4	669	54.5
	Graduate degree	215	16.1	204	18.9	242	19.7
**Gender identity**	Woman	626^1^	47.0	609^2^	56.6	622^4^	50.8
	Other	705	53.0	468	43.5	602	49.2
**Generation**	Gen Z	168	12.6	162^3^	15.0	187	15.2
	Millennial	566	42.3	467	43.3	443	36.1
	Pre-millennial	604	45.1	449	41.7	596	48.6

^1^For Gender identity in Survey 1, n = 1331 as a result of 7 missing values. Statistics for models incorporating this variable were adjusted accordingly.

^2^For Gender identity in Survey 2, n = 1077 as a result of 3 missing values. Statistics for models incorporating this variable were adjusted accordingly.

^3^For Generation in Survey 2, n = 1078 as a result of 2 missing values. Statistics for models incorporating this variable were adjusted accordingly.

^4^For Gender identity in Survey 3, n = 1224 as a result of 3 missing values. Statistics for models incorporating this variable were adjusted accordingly.

## Survey 1: Source of information

### Quantitative results

#### Structural equation model

**Fig 2:**
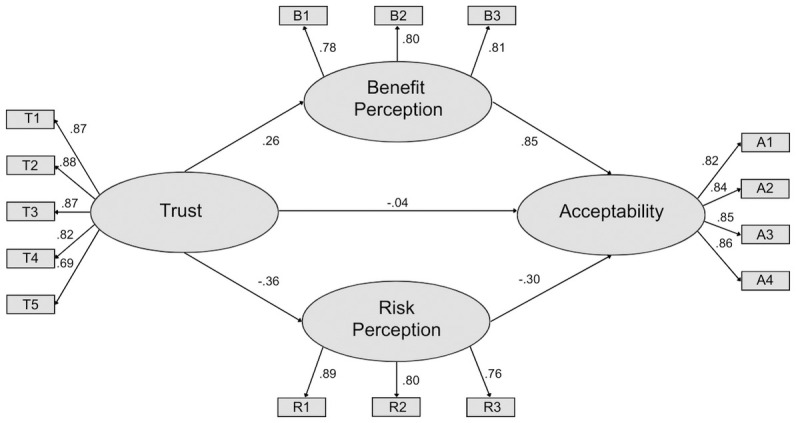
Social acceptance model for Survey 1. Values represent standardized estimates. All coefficients are significant (p < 0.001) with the exception of a tendency for the direct influence of trust on acceptance (P = 0.06).

The model fit for Survey 1 (Fig 2) was good as assessed by CFI (0.97) and adequate as assessed by RMSEA (0.063). We had hypothesized that social trust would directly influence acceptance, but this relationship was only a tendency (P = 0.06). All other hypothesized causal pathways were confirmed (P < 0.0001). Perceived benefit had the greatest effect on social acceptance. Social trust had an indirect influence on acceptance through effects on perceived benefit and perceived risk. Perceived benefit had a positive and perceived risk had a negative effect on acceptance.

#### General linear model

*Benefit*. Treatment did not affect participants’ perception of the benefit of the technology (gene editing) (F_14,1305_ = 1.25; P = 0.23); however, area (F_1,1305_ = 6.91; P = 0.009), country (F_1,1305_ = 7.35; P = 0.007), gender identity (F_1,1305_ = 22.24; P < 0.0001), and generation (F_2,1305_ = 10.90; P < 0.0001) were all significant; participants living in rural areas, living in Canada, women, and pre-millennials had lower perceptions of benefit. Education (F_5,1305_ = 7.00; P < 0.0001) also had an effect on perceptions of benefit, driven by participants with bachelor and graduate degrees perceiving the technology to have more benefit. No other demographics were significant.

*Risk*. Perceptions of risk of the technology were affected by the source of information (F_14,1305_ = 1.90; P = 0.02), with participants perceiving the use of the technology to be less risky when its use was proposed by private industry scientists (means ± SE; 2.4 ± 0.09) compared to when it was proposed by either environmental advocates (2.9 ± 0.10), grocery stores (2.8 ± 0.09) or dairy industry scientists (2.8 ± 0.09). Country (F_1,1305_ = 4.72; P = 0.03) and gender identity (F_1,1305_ = 4.68; P = 0.03) had effects on participants’ perceptions of risk of the technology, with Canadian participants having lower and women having higher perceptions of risk. All other demographic variables were not significant.

*Social trust*. Perceptions of social trust around the use of the technology tended to be affected by the source of information (F_14,1305_ = 1.58; P = 0.08). Social trust was highest in dairy veterinarians (mean ± SE; 2.5 ± 0.09) and lowest in grocery stores (2.1 ± 0.09). Generation (F_2,1305_ = 5.06; P = 0.007; driven by Pre-millennial participants reporting lower perceptions of social trust) and gender identity (F_1,1305_ = 3.85; P = 0.05; with women having lower perceptions of social trust) were the only significant demographics (see Table Survey 1 model statistics in Supplemental Material; https://doi.org/10.5683/SP3/NX3LZ9).

### Qualitative results

#### Source of information

Participants discussed sources of information in relation to their expertise or who they worked for. For instance, an animal scientist was usually described as someone who studied animals, specifically animal genetics and farm animal production. Government scientists were described as a scientist employed by the government. Likewise, participants described dairy farmers and dairy producers similarly and sometimes used terms interchangeably. In the words of one participant: “I would define [dairy producer] as a farmer or company that produces various types of dairy” (US1_457). Similarly, dairy processors were considered to be similar to dairy farmers in that they produced milk on a farm: “A person who works on a dairy and milks the cows” (US1_353). Finally, participants described advocates as defending and protecting an entity such as animals or the environment.

A few participants provided more elaborate responses. For example, some in the government scientist treatment felt scientists were controlled by the government and thus had compromised morals: “A scientist who works for the government and whose job is to do whatever the government is telling them to do even if it may not necessarily be the most morally correct” (US1_763). In contrast, some described private industry scientists as a source of information not being influenced by the government or politics: “A scientist who is mainly concentrating on a particular industry or issue centered around a private company or entity. Not regulated by governmental concerns” (US1_918). Similarly, some participants referred to dairy producers as corporations motivated by profit, for example: “Probably a big corporation, not some family farm. Farmers are under the boot of capital” (CA1_135).

#### Gene editing

Participants understood gene editing in three main ways: 1) a genetic change in an organism, 2) a genetic change that improves an organism, or 3) a genetic change in an organism that involves technology. Participants who understood gene editing as a genetic change responded simply that gene editing changes the DNA of an organism and interchanged verbs like “modifying”, “altering”, “editing”, “splicing”, “adding”, and “deleting”. In a small number of cases, participants interchanged or associated other terms with gene editing; for example: “Gene editing is essentially genetic modification” (US1_288) or: “Gene editing is a fancy word for altering DNA” (US1_69). Some participants believed gene editing to be beneficial, referring to “specific benefits”, “improved traits or outcomes” or “removing bad/defective genes”. For example, one participant responded that gene editing is: “Changing the genes of an animal or plant to help adapt to changes in the environment” (US1_692). Finally, some participants referred specifically to technology involved with gene editing, and used the terms like “science”, “laboratory” and “CRISPR” in their responses, including, for example: “gene alterations in laboratories” (US1_630) and: “modifying the genome of a living organism using some technologies” (CA1_34).

Some participants expressed other understandings. For example, some described gene editing as “unnatural”, “artificial” or “abnormal”, or expressed caution about the technology. For instance: “It is unnaturally changing the very DNA of animals to possibly harm or affect others without the intention of doing so, yet only looking at the short-term possibilities instead of the entire ecosystem at large” (US1_70). Others noted that gene editing was done for human “desires”, “needs”, “wants” and “benefits”. For example, one participant wrote: “I think it’s where genes are changed to fix a problem or to change a situation in which a company or person wants” (US1_916). A small number understood gene editing to be an example of selective breeding or crossbreeding (i.e. breeding done with actual animals, not on a cellular level). Finally, a few participants stated they did not understand what gene editing was.

## Survey 2: Technology term

### Quantitative results

#### Structural equation model

**Fig 3:**
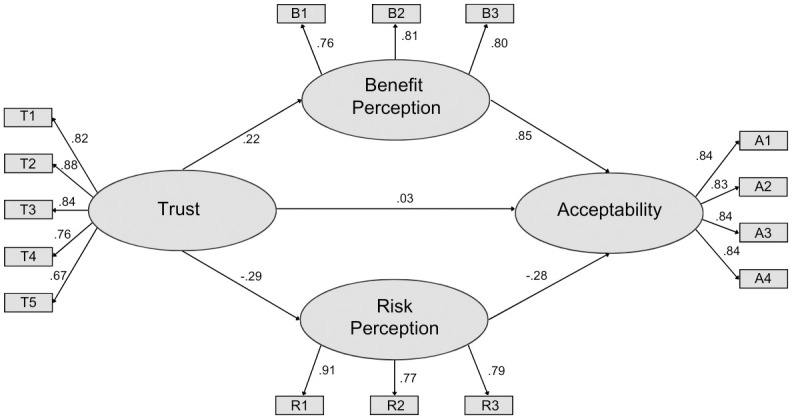
Social acceptance model for Survey 2. Values represent standardized estimates. All coefficients are significant (p < 0.001). There was no evidence of a direct influence of trust on acceptance (P = 0.21).

The model for Survey 2 (Fig 3) obtained a good fit (CFI = 0.96; RMSEA = 0.064). Similar to Survey 1, we hypothesized that social trust directly influenced acceptance, but our results showed no evidence of this relationship (P = 0.21). All other hypothesized causal pathways were significant (P < 0.0001). Like Survey 1, perceived benefit had the greatest effect on acceptance. As well, social trust had an indirect influence on acceptance through effects on perceived benefit and perceived risk, with perceived benefit and perceived risk having positive and negative effects on acceptance, respectively.

#### General linear model

*Benefit*. Treatment (technology term) did not affect participant perceptions of the benefit of the technology (F_7,1056_ = 1.29; P = 0.25), but country (F_1,1056_ = 5.26; P = 0.02), education (F_5,1056_ = 9.49; P < 0.0001), and gender identity (F_1,1056_ = 15.57; P < 0.0001) each had an effect. Canadian participants and women perceived the technology as having lower benefit; the effect of education was driven by participants with bachelor and graduate degrees perceiving the technology as more beneficial.

*Risk*. Perceptions of risk of the technology were not affected by the technology term (F_7,1056_ = 1.23; P = 0.28), but were affected by gender identity (F_2,1056_ = 17.39; P < 0.0001; with women perceiving the technology as having higher risk). Other demographics had no effects on perceived risk around the use of the technology.

*Social trust*. Neither treatment (F_7,1056_ = 1.15; P = 0.33) nor any of the demographic factors affected perceptions of social trust around the use of the technology (see Table Survey 2 model statistics in Supplemental Material; https://doi.org/10.5683/SP3/NX3LZ9).

### Qualitative results

Participants understood the technology terms in the same way as they did in Survey 1 where, regardless of the specific term used, the technology involved a change in an organism’s DNA. Participants also interchanged verbs and terms to describe the technology in their responses. For example: “To me, that essentially means "genetic modification" (US2_757; genetic alteration); “genetic engineering or genetic modification” (US2_602; genetic manipulation); “Gene editing is similar to genetically modifying produce. Desirable qualities are added to organisms” (US2_25; gene editing).

As observed in Survey 1, a few participants expressed other understandings across technology terms including feeling cautious about the technology or considering the technology to be unnatural. Caution typically related to unknown consequences and side effects. For example: “It is very risky and can have unintended side affects” (US2_499; DNA modification); “I am unsure of long-term effects on the cows and consumption to humans” (US2_92; gene editing). Concerns about naturalness also arose. For example: “I do not know the process, only that it is trying to "fool mother nature" (US2_669; genetic manipulation); and “Not natural, fake, chemicals and other alternatives are entered into the equation” (US2_366; genetic engineering).

## Survey 3: Application of the technology

### Quantitative results

#### Structural equation model

**Fig 4:**
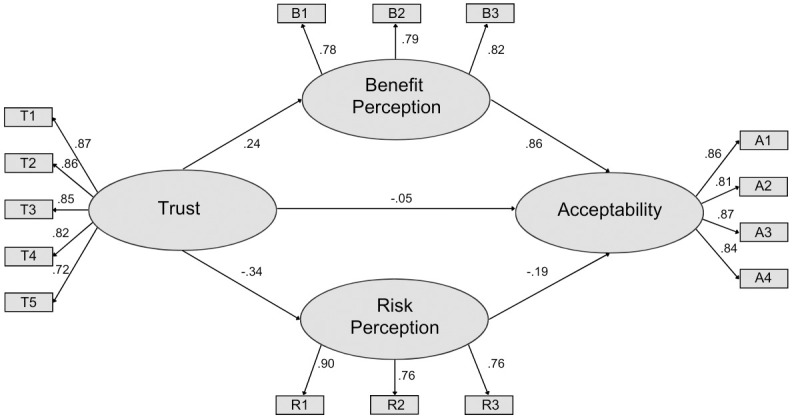
Social acceptance model for Survey 3. Values represent standardized estimates. All coefficients are significant (p < 0.001) with the exception of the direct effect of trust on acceptance (P = 0.03).

The model for Survey 3 (Fig 4) obtained a good fit (CFI = 0.96; RMSEA = 0.065). For Survey 3 we found a direct effect of social trust on acceptance (P = 0.03), although this effect was modest compared to all other hypothesized causal pathways (P < 0.0001). The model for Survey 3 was similar to that for Survey 1 and Survey 2; perceived benefit again had the greatest effect on social acceptance, and social trust also had an indirect influence on social acceptance through effects of perceived benefit and perceived risk.

#### General linear model

*Benefit*. Participants’ perceptions of the benefit of the use of the technology were affected by the application (F_4,1207_ = 2.93; P = 0.02). Overall, applications involving animals were perceived as less beneficial than the application involving plants (F_1,1207_ = 9.36, P = 0.002). Table 4 provides mean and SE for the perceived benefit, perceived risk and social trust for each application. Country (F_1,1207_ = 20.60; P < 0.0001), education (F_5,1207_ = 11.91; P < 0.0001), and generation (F_2,1207_ = 28.40; P < 0.0001) affected perceptions of benefit; Canadian and Pre-millennial participants perceived the technology as having lower benefit, and the effect of education was driven by participants with bachelor or graduate degrees perceiving the technology as more beneficial.

**Table 4:** Mean and standard errors for the latent variables (calculated using path coefficients from SEM Fig 4) of perceived benefit, perceived risk, and social trust for each application in Survey 3.

	Perceived benefit	Perceived risk	Social trust
**Application**			
**Cattle heat stress resistance**	2.6 ± 0.04	2.7 ± 0.04	2.3 ± 0.04
**Wheat plant drought resistance**	2.8 ± 0.05	2.6 ± 0.06	2.5 ± 0.06
**Pig disease resistance**	2.6 ± 0.05	2.6 ± 0.06	2.5 ± 0.06
**Allergen-free milk**	2.6 ± 0.05	2.6 ± 0.05	2.4 ± 0.06
**Increased muscle growth in cattle**	2.6 ± 0.05	2.8 ± 0.06	2.3 ± 0.06

*Risk*. Perceptions of the risk were also affected by the application (F_4,1207_ = 3.29; P = 0.01), driven by higher perceptions of risk in the increased cattle muscle application and lower perceptions of risk in the wheat plant mildew resistance application. Applications involving animals tended to be perceived as more risky than the application involving plants (F_1,1207_ = 2.89, P = 0.09).

Country (F_1,1207_ = 9.10; P = 0.003) and gender identity (F_1,1207_ = 18.76; P < 0.0001) affected perceptions of risk, with Canadian participants having lower perceptions of risk and women having higher perceptions of risk, respectively. Education (F_5,1207_ = 2.62; P = 0.02) also affected perceptions of risk, driven by participants with bachelor and graduate degrees perceiving the technology to involve more risk.

*Social trust*. Participants’ perceptions of social trust around the use of the technology were also affected by the application (F_4,1207_ = 2.51; P = 0.04); this difference was driven in part by the tendency to view the applications involving animals more negatively than the application involving plants (F_1,1207_ = 3.72, P = 0.05). Demographics had no effect on participants’ social trust (see Table Survey 3 model statistics in Supplemental Material; https://doi.org/10.5683/SP3/NX3LZ9).

### Qualitative results

We categorized participant responses about the acceptability of the technology application into three themes: 1) impacts on plants, animals, and people, 2) trust in sources of information (i.e. actors) and technology, and 3) ethical approaches.

#### Impacts on plants, animals, and people

Participants discussed pain and suffering in animals, the necessity of gene editing, and comparisons of gene editing to other practices when considering the acceptability of the technology. While some participants believed that gene editing would cause suffering: “I think it is unacceptable. Who knows what other things can go wrong or how the pigs could suffer from being genetically modified?” (CA3_77; pig disease), others felt that gene editing could alleviate suffering: “The animals will be able to survive the heat better and won’t suffer as much. This gives the animals a better quality of life in the aspect of heat” (US3_236; cattle heat resistance). Some participants based their acceptance of gene editing on whether it might cause suffering; for example: “It would depend on whether or not the animals are suffering. Does "heat tolerant" simply mean that the cow can survive biologically? If it survives, but is suffering, the technology is not [acceptable]” (US3_23; cattle heat resistance).

Addressing societal problems, including food production, was another topic. As one participant offered: “I think that anything that helps increase crop yield is a net benefit for society. We are rapidly running out of room and are increasing our population to the point where we need to consider all options when it comes to being able to feed our population” (US3_741; wheat mildew). In contrast, some participants felt that gene editing was a “band-aid” for larger problems: “I believe genetically altering the dairy cows may fix the problem in the short run. However, if we don’t address the root causes of climate change, then the dairy farmers will have to face this problem again in the future” (US3_47; cattle heat resistance). Some participants questioned the necessity of using gene editing to address the problem presented in the vignette. As one participant argued: “… There are so many milk alternatives available. There is no need to use this technology” (US3_462; allergen-free milk).

Participants also drew comparisons between gene editing and other agricultural practices, processes done to humans, and selective breeding. Some participants suggested that the technology was similar to others, for example: “If we have accepted all the other technologies in modern livestock farming, I don’t think this is significantly different” (US3_225; cattle heat resistance). Others felt that gene editing was similar to selective breeding, and this similarity increased acceptability: “I don’t see anything inherently wrong with the idea of genetic modification of crops to improve things like yield or resistance to diseases. After all, it’s essentially doing the same thing that humans have done for millennia by selective breeding, only now it can be achieved much more quickly” (US3_799; wheat mildew). In contrast, another participant discussed how the end result would be acceptable if done through selective breeding, but not if achieved with the technology proposed (i.e. through genetic modification): “If this is done through selective breeding then I would consider it more acceptable, but if done through other means it is inappropriate” (CA3_12; cattle heat resistance). Finally, a few participants that were in either the wheat mildew resistance or pig disease resistance treatments compared gene editing to human vaccines: “This is similar to vaccines, where injecting them helps humans evolve from this disease and become more resilient” (CA3_94; wheat mildew).

#### Trust in sources of information and technology

Participants expressed whether they felt the groups involved in gene editing were trustworthy and related this to acceptability. Certain groups were discussed as trustworthy: “I rely on the expertise of the farmers and scientists to determine the effect on the plants and environment and also rely on their judgment on whether or not to use it and how widely it should be used. So, I think it is acceptable” (US3_811; wheat mildew). However, other groups were discussed as not to be trusted, with reasons often associated with concerns regarding underlying profit motives. As one participant described, “Farmers want more profit, and their decisions will be based on personal finances. They cannot be trusted to make ethical decisions when it comes to genetically modifying animals, or its effects on the environment” (US3_616; pig disease). Sometimes distrust was directed at large businesses, including large farms: “… the big farms are driven by money like any big corporation. Financial motivation has historically caused bad decisions to be made at the risk of the health and welfare of people and the environment” (US3_428; allergen-free milk). Similarly, another participant commented: “When I think of the ‘farmer’ I think of Ma and Pa […], but we all know that ‘farmer’ doesn’t mean Ma and Pa […]. Instead, ‘farmer’ refers to Perdue and the other Big Agriculture companies out there. While these companies may have the intellectual ability to properly assess and evaluate genetically modified foods and their impact on humanity and the environment, their intellect is completely blinded by the profit margin. I have no faith in these corporations at all” (US3_812; wheat mildew).

Participants often expressed concern about unforeseen consequences of gene editing. Concerns were raised regarding consequences to the animal subjects and to those consuming the food products: “While it might feed more people, we do not know the long-term effects it could have on animals, nor the long-term effects on the people consuming the product” (US3_342; increased cattle muscle growth). Others were concerned about the environment and natural world, for example: “I don’t think it’s acceptable, because no one knows the long-term effects that genetically modifying plants will have on the environment” (US3_729; wheat mildew) and “Most times, when humans have tried to solve problems by altering nature, it has had negative consequences” (US3_20; cattle heat resistance).

A related topic focused on transparency and ensuring the technology was thoroughly researched and regulated. For instance, participants wrote: “I think that the technology needs to be thoroughly tested by qualified researchers. I also think that the use of the technology should be made transparent to everyone involved from the farmer all the way down to the consumer” (US3_489; allergen-free milk) and “I worry greatly about use of this technology without a great deal of regulation” (US3_124; cattle heat resistance).

#### Ethical approaches

Some participants approached the acceptability of gene editing using a deontological approach and felt that gene editing was immoral under any circumstance and if used, would create an ethical “slippery slope” leading quickly to other contentious practices. In the words of one participant: “I don’t think it’s morally right to genetically modify animals to fit our needs. I think this opens the door to way more things that are even more morally wrong” (US3_135; cattle heat resistance). Other participants expressed a specific opposition to using animals in gene editing: “I don’t believe technology should be used upon animals whatsoever. It doesn’t matter the reasoning as to why. If it’s not something that directly benefits the actual animal, it shouldn’t be done” (US3_297; increased cattle muscle growth). One participant felt that gene editing violated animal rights: “I feel it clearly violates animal rights laws that are in place to protect animals in general from a movement such as this” (US3_430; allergen-free milk).

Others took a utilitarian approach and weighed risks and benefits surrounding the use of the genetic technology. One participant wrote: “The respiratory syndrome in particular is probably very distressing to the pigs and eliminating it as a health concern makes me want to lean towards approving of the solution in this situation. There are risks. However, I think that the benefits outweigh those risks with this particular animal health issue” (US3_587; pig disease). Others felt that benefits did not outweigh risks: “It is not acceptable at all. Who knows what problems will arise farther down the road? Short term benefits do not outweigh long term problems” (US3_144; cattle heat resistance). Finally, some participants demonstrated mixed feelings: “I’m on the fence about it. I don’t like the idea of using genetically modified anything. It just doesn’t seem natural. But then again, if it helps farmers and people survive, maybe it’s ok and should be considered” (US3_819; wheat mildew).

## Discussion

Model fit for Surveys 1, 2 and 3 was good and the latent variables of perceived benefit, perceived risk and social trust had similar effects on acceptance in each survey. Perceived benefit had the greatest effect on acceptance and social trust had an indirect influence on acceptance through effects of perceived benefit and perceived risk. Contrary to our hypothesized model, social trust had little direct influence on acceptance. The proposed application of the technology affected perceptions of perceived benefit, perceived risk, and social trust, but the source of information and the term used to describe the technology had little effect.

In Survey 1, we presented participants with 15 sources of information. Our approach differed slightly from previous research that asked participants to express their trust in institutions or broad categories of individuals responsible for GE [16,18], sometimes including specific companies [19]. There are several approaches to conceptualizing trust, including examining the relationship between trust and heuristics as well as comparisons to related concepts like confidence and expertise [17]. Future research examining how sources of information influence social acceptance of GE may wish to explore other models of trust.

The majority of participants described the technology terms as referring to a change in DNA and in some cases used different terms interchangeably. This contrasts with previous work that has examined how members of the public perceive GE. One previous study on US participants found that “genetic modification” had the lowest perceived benefits, positive affect, support and purchase intention, and that “agricultural biotechnology” had higher perceived benefits, support and purchase intention [21]. A different study on Dutch participants found that the terms “genomics” and “genetic modification” resulted in more negative attitudes compared to “natural crossing” and “traditional breeding” [20]. Lithuanian survey participants were less negative about food produced through “gene editing” versus “gene modification” [22]. Another study, this time on Swiss and British participants, found a similar difference when comparing “genetically-modified tomatoes” discussed in a scientific newspaper versus “gene-edited tomatoes” discussed in a blog [23]. Finally, [55] found that participants from five different countries stated that they were more willing to consume “CRISPR” rice than “GMO” rice. The terms used to describe the technology are debated in the regulatory sphere [56–58], suggesting that stakeholders perceive this to be important. Comparisons between cis-genetic and trans-genetic applications also appear in these discussions [see 59,60 for two recent examples]. Taken together these previous studies suggest that the way in which terms are defined and presented can affect perceptions, but our work indicates that in some cases at least this effect is likely to be small, and that many people recognize the terms as synonymous.

In Survey 3, animal applications were perceived as less beneficial than plant applications, a finding that has been previously reported [12,25,26,32]. Participants in our study described the acceptability of the technology based on how it could cause or alleviate suffering in animals, solve problems with food production and whether they trusted the technology and individuals involved. Perceived risk for the increased cattle muscle growth application was slightly higher than that for the other applications (Table 4), perhaps driven by differences in the acceptability of different applications. Perceived risk of the muscle growth application may tie in with views around the beneficiary of the technology; earlier work has shown that GE used for economic gain is viewed as less acceptable than applications that provide some public good [25,61]. Participants also approached the question from ethical and regulatory perspectives often weighing benefits and risks or defining their expectations regarding appropriate technology use. These findings are similar to those from previous research, including concerns about animal welfare, ethical considerations and uncertainty about the technology and how to control it [29,33], as well as weighing benefits and risks and trustworthiness of the technology [31]. Earlier work has reported concerns with unknown long-term consequences and acknowledged that the technology may benefit farmers, consumers, and others [62]. Others have reported concerns including animal welfare, dignity and standards of care [63].

Given that perceived benefit had the greatest effect on acceptance in this study, we encourage future work to consider perceptions of benefit in more depth. This might be done by considering whether proposed benefits to animal welfare through GE are actually seen as beneficial to animals by the public [64], whether or not proposed or promised benefits are realized in the application, and evaluating how different publics define “benefit”. Further, “benefit to animals” could be added as an additional observed variable in the model to determine how benefit to animals is perceived and how this contributes to the perceived benefit construct. Finally, our study focused on cattle but did not specify whether cattle were used for beef or dairy farming; future work might investigate this and other farming contexts. We included one application for pigs and this application was not perceived as more beneficial or risky compared to the others. Detailed cases describing species and farm context might provide more insight beyond the broad categories sometimes considered in earlier research (e.g., valuation of “GM meat”; 11).

Our findings correspond with earlier work showing that risk and benefit perceptions are inversely related [see 65]. Given the complexity of risk and benefit perceptions, understanding the weight people apply to each of these is important [66]. Our findings showed that perceived benefit of the technology had the greatest effect on acceptance, although risk perceptions and the indirect effect of social trust were also important.

In each survey, the observed variable T5 (“*I feel confident that the [source of information] acts without political or private pressures and obligations when assessing this technology”)* was the poorest indicator of social trust (Survey 1 = 0.69; Survey 2 = 0.67; Survey 3 = 0.72), perhaps because this question addresses the integrity of institutions rather than their competence [34]. Future work might consider how recent democratic political processes have challenged social trust in institutions, perhaps also considering the concept of distrust [67].

The causal model developed by Siegrist [16,35] has been used to investigate acceptance of other novel technologies [see 68], and our results illustrate the power of this model in accounting for variation in social acceptance of GE technology with farm animals. Most research about GE acceptance has been completed in North America and Western Europe, and little is known about how acceptance models would function in other areas of the world [68]. To this point, future research should also extend beyond perceived benefit, perceived risk, and social trust and further explore the economic, political and situational contexts that shape these perceptions [65]. Finally, we suggest that inquiries begin by aiming to understand the actual concerns expressed by research participants, for example in focus groups [e.g. 69], through in-depth contextual understandings [63] or other constructs that examine core values [e.g. moral foundations; 25].

This study has several limitations. First, our use of the causal model focused on perceived benefits, perceived risk, and social trust which could have primed participants to discuss these concepts and use this terminology in their open-ended responses. Indeed, in Survey 3, participants discussed trust in sources of information (i.e. actors), weighed benefits and risks of GE, and considered whether GE was acceptable to be used to solve larger societal problems (e.g., environmental, hunger). Although participants brought up specific examples and other concerns (e.g. ethics, absolute opposition), their responses could have been shaped by our questions. Second, the open-ended questions in Survey 1 and 2 did not result in nuanced responses about how participants understood the source of information or technology terms. For example, approximately 15% of responses in both Survey 1 and 2 were coded as “not applicable” because answers were short (e.g. “good”) or participants wrote “I understand”, perhaps signifying that they misinterpreted the question.

Our vignettes may have introduced limitations. Participants responded to open-ended questions after they read the vignette that described how the technology would make cattle more heat resistant, which, given the positive framing, could have led participants to mention improvements for animals (one of our results). One of our aims in asking these questions was to determine how and whether people understood the terms, however the majority of the responses were answered in just a few words. Our vignettes specified ‘cattle’ and did not provide specific information about the use of these animals (e.g., dairy, beef); we are unsure whether these details would have added helpful information for survey participants. Additionally, our original vignette presented temperature only in Celsius which may have confused some US participants.

While our SEM was sufficiently powered, our analysis of treatment effects should be interpreted with caution because the number of treatments in each survey, especially Survey 1 (15 treatments) and Survey 2 (8 treatments), resulted in small within-cell samples. Finally, while there are numerous benefits of MTurk (e.g., large participant pool, ease of data collection, cost), we acknowledge that samples may not be representative and other data quality issues may arise [49]. To improve data quality, we developed a multi-step screening process based on the assessment of qualitative, open-ended text responses.

One methodological innovation in the current study was our use of Turnitin to screen open-ended responses, allowing us to identify responses that participants copied from online sources. The copied responses could be considered lower quality, so our method provides a new form of quality control for online surveys. In addition, these copied responses provided us with data on which online sources were used by participants to research questions about GE. Previous research has assessed the information about GE in print and digital media [e.g. 70,71] and social media [e.g. 72,73], but never directly assessed what sources participants refer to when provided the opportunity.

## Conclusions

In three separate surveys we used a causal trust-acceptability model to investigate social acceptance of GE technologies in farm animals (mainly cattle). Perceived benefit of the technology had the greatest effect on acceptance. Participants understood that GE technologies changed an organism’s DNA, but were not greatly influenced by the specific term used to describe this technology or the terms used to describe the proponent of the technology. Participants described the acceptability of applications by considering the impact GE can have on plants, animals, and people, including on societal problems like global food production. The powerful explanatory properties of this model suggest that future studies should focus on how applications developed for different animals and perceptions of benefits for animals specifically relate to perceptions of benefit, risk, and social trust.
